# Hyponatremia With Anticonvulsant Medications: A Narrative Review

**DOI:** 10.7759/cureus.57535

**Published:** 2024-04-03

**Authors:** Kristin Nicole Bembenick, Jibin Mathew, Michael Heisler, Harish Siddaiah, Peyton Moore, Christopher L Robinson, Adam M Kaye, Sahar Shekoohi, Alan D Kaye, Giustino Varrassi

**Affiliations:** 1 School of Medicine, Louisiana State University Health Sciences Center, Shreveport, USA; 2 Department of Anesthesiology, Louisiana State University Health Sciences Center, Shreveport, USA; 3 Department of Anesthesiology, Critical Care and Pain Medicine, Beth Israel Deaconess Medical Center, Harvard Medical School, Boston, USA; 4 Department of Pharmacy Practice, Thomas J. Long School of Pharmacy and Health Sciences University of the Pacific, Stockton, USA; 5 Pain Medicine, Paolo Procacci Foundation, Rome, ITA

**Keywords:** epilepsy, seizures, adh, aeds, sodium, hyponatremia

## Abstract

Hyponatremia is an adverse effect of many antiseizure medications (ASMs). It occurs with interference with the normal balance of electrolytes within the body. Various risk factors associated with the development of hyponatremia in patients taking these medications include age, gender, dosage, and combinations with other drugs. ASMs such as carbamazepine (CBZ), oxcarbazepine (OXC), and valproic acid have a higher risk of hyponatremia. Hyponatremia induced by an antiseizure medication can occur through various mechanisms depending on the drug's specific mechanism of action. Hyponatremia can be a potentially fatal side effect. Patients taking these medications need to be monitored closely for the signs and symptoms of hyponatremia. Acute hyponatremia, defined as developing in <48 hours, is more likely to show symptoms than chronic hyponatremia. Signs of acute hyponatremia include delirium, seizures, decerebrate posturing, and cerebral edema with uncal herniation. Chronic hyponatremia, defined as developing in >48 hours, can cause lethargy, dizziness, weakness, headache, nausea, and confusion. Hyponatremia is associated with longer hospital stays and increased mortality. Treatment varies based on the degree of severity of hyponatremia. Choosing a treatment option should include consideration of the drug causing the electrolyte disturbance, the patient’s risk factor profile, and the severity of symptoms as they present in the individual patient. Healthcare providers should be aware of hyponatremia as a potential side effect of ASMs, the signs and symptoms of hyponatremia, the different treatment options available, and the potential complications associated with rapid correction of hyponatremia.

## Introduction and background

Hyponatremia is the most common electrolyte disorder and is characterized by a sodium level <135 mEq/L [1]. It affects hospitalized patients at a greater rate than the general public. A low serum level of sodium can cause headache, fatigue, anorexia, vomiting, seizures, and coma [2]. The symptoms are due to the low serum sodium level pulling the excess water from the bloodstream into the cells including the brain tissue [2]. The severity of side effects often correlates to the degree of hyponatremia. However, even mild hyponatremia is associated with increased hospital stay length and mortality [1].

Hyponatremia is a known adverse effect in patients on various drugs, including patients using anticonvulsant drugs, otherwise known as antiseizure medications (ASMs) [2]. ASMs are a group of medications commonly used to treat epilepsy and other seizure disorders [3]. ASMs work through various mechanisms [4]. Consideration in ASM choice must include looking at the spectrum of efficacy, pharmacokinetic characteristics, safety and tolerability, efficacy against comorbidities, and each drug's indications relevant to the patient's circumstances (4). ASMs may be divided into different groups depending on their mechanism of action. The most common groups include drugs that facilitate gamma-aminobutyric acid (GABA) ergic neurotransmission and those that block neuronal ion channels [5]. This does not encompass every ASM, and some of the newer drug's mechanisms of action are currently being studied [5]. Although hyponatremia can be caused by almost all ASMs, it is most commonly seen in drug groups that block neuronal ion channels, specifically those that act on sodium channels [6].

There are several mechanisms by which ASMs can lead to hyponatremia [7]. One common cause of hyponatremia is an increased secretion of antidiuretic hormone (ADH), which promotes water retention, diluting the sodium concentration in the blood [7]. ASMs were found to cause hyponatremia via an increased sensitivity to circulating ADH [8]. In most cases, the hyponatremia caused by ASMs is transient and asymptomatic.

Acute hyponatremia (defined as developing a serum sodium level <135 meq/L in <48 hours) is more likely to be symptomatic than chronic hyponatremia. Delirium, seizures, decerebrate posturing, and cerebral edema with uncal herniation are symptoms more likely observed with acute hyponatremia. Chronic hyponatremia (developing in >48 hours) can cause lethargy, dizziness, weakness, headache, nausea, and confusion [6]. Hyponatremia associated with carbamazepine (CBZ) and oxcarbazepine (OXC) is more likely to be the chronic type [6]. Previous research has shown that risk factors for hyponatremia in patients on ASMs include age, baseline sodium level, dose or blood concentration of the drugs, and concomitant use of other ASMs, including levetiracetam. Concomitant drugs that are not ASMs may also increase the risk of hyponatremia, including diuretics. Other factors that can contribute to hyponatremia in people taking ASMs include volume-depleting conditions such as dehydration, vomiting, diarrhea, and excessive sweating. These conditions can exacerbate an imbalance in electrolytes, including sodium [2].

It is important to monitor sodium levels in people taking ASMs. Healthcare workers need to be aware of the symptoms of hyponatremia to identify the electrolyte imbalance and treat it accordingly [6]. Treatment for hyponatremia may involve adjusting the dose of the ASM, managing fluid intake, restricting fluids, and correcting any underlying medical conditions contributing to the imbalance in sodium levels [9]. In this review, we will discuss common ASMs associated with hyponatremia, the mechanism of action of various ASMs, clinical symptoms, the presentation of hyponatremia, and treatment strategies for hyponatremia in patients using ASMs.

## Review

Methods

This is a narrative review. The sources for this review are as follows: searching on PubMed, Google Scholar, Medline, and ScienceDirect using keywords epilepsy, seizures, ADH, AEDS, sodium, hyponatremia.

Antiepileptic drugs

Epilepsy is one of the most common neurologic disorders, occurring throughout the age span. It is found in approximately 1% of the general population [10]. The first line of treatment for epilepsy is anti-seizure drugs, also known as ASMs or anticonvulsant medications. ASM development has rapidly accelerated in the last three decades with the creation of the US National Institutes of Health (NIH)/National Institute of Neurological Disorders and Stroke (NINDS)-sponsored Anticonvulsant Screening Program (ASP), later changed to the Epilepsy Therapy Screening Program (ETSP) along with advances in our knowledge of the neurobiology of epilepsy [10]. Since the ASP and ETSP creation, 18 new ASMs have been approved for clinical use. The newer drugs possess more favorable pharmacokinetic profiles that act on various molecular targets; however, there has been no significant improvement in the therapeutic efficacy of the drugs [10].

Before the 19th century, the treatment of epilepsy was based largely on religious or supernatural beliefs. The condition was initially presented with a negative perspective, often with the patients suffering from persecution and discrimination. The treatment consisted primarily of herbal remedies, organic concoctions, and chemicals that lacked respect for scientific scrutiny [10]. Hippocrates described epilepsy as a brain disorder circa 460-370 BC. It was not until the late 1700s to mid-1800s that his theory took root [10]. The first treatment came in 1857 from Sir Charles Locock, who recognized that various inorganic bromide salts resulted in sedative effects [10]. Potassium bromide remained the de facto treatment for epilepsy until 1912 when phenobarbital became available after the discovery of its sedative effects on dogs [10]. To this day, phenobarbital remains an effective treatment for epilepsy, commonly used in infants and developing countries. In the 1930s, phenytoin was discovered after Merritt and Putnam invented the electroshock threshold test to be used with cats. Phenytoin proved to be more effective and less sedating than either potassium bromide salts or phenobarbital [10]. At the beginning of the NINDS ASP, despite many efforts to develop multiple new drugs, the only drug developed and approved for use in the US was ethosuximide. Between the 1960s and mid-1970s, valproate and CBZ were developed in Europe and only later brought to the US. The first clinically available benzodiazepine, chlordiazepoxide (Librium), also became available in the UK, followed shortly by diazepam (Valium) [10]. It became evident that the progress in the US by the NINDS was painstakingly slow. It was not until around 1990 that the NINSD ASP began producing many new drugs, categorized as third-generation ASMs [10]. Despite the efforts put into developing new and improved ASMs, the side effect profile remains large and often deters patients from complying with their medication.

Adverse effects and challenges associated with ASMs

Commonly reported side effects associated with ASMs include feelings of tiredness, upset stomach, dizziness, sexual dysfunction, urinary retention, and blurred vision [11]. Many of these happen within the first weeks of taking the medications. Patients also report psychological problems associated with slower thinking, trouble remembering or paying attention, and finding the right words to use [11]. Many of these adverse effects lead patients to stop using the medication shortly after starting it.

ASMs are known to be teratogenic, leading to possible miscarriages and congenital malformations [12]. However, the prevalence of congenital malformations seen with ASM monotherapy declined from 6.1% in 1998-2004 to 3.7% (−39%) in 2015-2022, most likely due to the increased use of levetiracetam during pregnancy which is associated with less of a risk. ASMs can also cause sexual dysfunction in both men and women. Many patients suffering from epilepsy are young, and these side effects deter them from being compliant with their medications. As the doctor, it is important to inform the patients of these adverse effects and their potential benefits, helping them appreciate the challenges associated with the medication. The use of ASMs is also related to social isolation, dependent behavior, low rates of marriage, unemployment, and reduced quality of life [11]. There is also an economic burden associated with ASM use. The new development of the medications has been costly, and they are still often not readily available in under-resourced healthcare areas [11]. As we will discuss in detail in this article, ASM's adverse effect profile also includes electrolyte abnormalities, with the most dangerous being hyponatremia. All challenges associated with medication compliance should be addressed, but the physician prescribing the ASM should also be acutely aware of the potential complication of hyponatremia by monitoring the patient's sodium regularly. If a patient develops symptoms of hyponatremia, the ASM should be stopped immediately and switched to an alternative medication.

Common anticonvulsants associated with hyponatremia

Hyponatremia, a condition defined by low levels of sodium in the blood, is a recognized adverse effect associated with various ASMs. Among these drugs, OXC and CBZ are the two most commonly linked to hyponatremia [13]. Eslicarbazepine (ESL) is structurally similar to CBZ and OXC and shares the same active metabolite as OXC [14,15]. Other ASMs such as phenytoin, topiramate, sodium valproate, and levetiracetam have also been reported to be associated with hyponatremia, albeit to a lesser extent than CBZ or OXC [16]. Gabapentin and pregabalin are less likely to cause hyponatremia, although a few isolated cases have been reported.

The association between CBZ, OXC, and hyponatremia dates back to the 1970s and 1980s, with varying reported incidences across different studies [17,18]. The incidence of hyponatremia caused by OXC ranges from 25-75%, while for CBZ, it varies from 5-40%. Studies have used different thresholds to define hyponatremia, contributing to this wide range. For instance, a cross-sectional study by Nielsen found the frequency of hyponatremia to be just over 50% in OXC-treated patients [18].

In a retrospective study by Friis et al., hyponatremia was noted in 23% of OXC-treated patients [19]. Another study by Dong et al. observed a higher frequency of hyponatremia in OXC-treated patients (29.9%) compared to CBZ-treated patients (13.5%) (p <0.0001). The incidence of severe hyponatremia (Na<128 mEq/L) was also higher in OXC-treated patients [20].

Data obtained from a pharmacogenomics database by Berghuis et al. revealed hyponatremia in one-fourth of those taking CBZ and nearly half of those taking OXC. Rates of severe hyponatremia were 22% and 7% for CBZ and OXC, respectively. Hyponatremia was symptomatic in 48% and led to hospital admissions in 3% [21]. A review of medical records by Intravooth et al. at a German epilepsy center showed that only CBZ, OXC, and ESL were correlated with hyponatremia. The incidence of hyponatremia induced by ESL was not significantly different from that induced by OXC (43% of patients with OXC and 33% with ESL, p>0.05). Both were correlated with hyponatremia more often than CBZ (16%). OXC-induced hyponatremia was dose-related, but ESL-induced hyponatremia was not [22].

The risk of hyponatremia with valproic acid, phenytoin, and topiramate is less understood and documented. In a retrospective cohort study by Gandhi et al., the use of valproate, phenytoin, or topiramate compared to no ASM use was associated with a 2.6-fold higher RR of hospitalization with hyponatremia within 30 days of drug initiation [16]. These findings run contrary to some studies where phenytoin was effectively used to reverse CBZ-induced water intoxication [23]. Hyponatremia from levetiracetam is rare, but the risk increases with polypharmacy, old age, and other risk factors [24,25].

Mechanism of action

The regulation of sodium homeostasis, intricately connected with water and osmotic balance, involves the coordinated action of the renin-angiotensin-aldosterone (RAAS) system and the ADH pathway. Hyponatremia, characterized by low sodium levels, typically results from a relative excess of water compared to sodium [7]. In the context of ASMs, hyponatremia has been predominantly linked to inappropriate hypersecretion of ADH or arginine vasopressin (AVP), leading to excessive water reabsorption and subsequent dilution of sodium levels.

Apart from stimulating AVP release, there is evidence to suggest that other mechanisms may cause hyponatremia from ASMs. Interestingly, individuals experiencing hyponatremia associated with CBZ or OXC use have shown both elevated and reduced levels of AVP. The term "Nephrogenic Diabetes Insipidus" was coined to describe a clinical presentation akin to the syndrome of inappropriate ADH secretion but with undetectable levels of AVP [26].

Studies conducted by Stephens et al. demonstrated impaired water handling in subjects receiving therapeutic doses of CBZ, with basal plasma AVP concentrations decreasing. This suggested that CBZ's water-retaining property is mediated by increased renal sensitivity to normal AVP concentrations and the resetting of osmoreceptors [8]. Similarly, investigations by Sachdeo et al. based on water loading tests after OXC intake concluded that OXC may directly affect renal collecting tubules or enhance their responsiveness to circulating AVP [27].

Experimental studies on rats by de Braganca et al. provided insights into the direct effects of CBZ on the renal collecting tubules. They demonstrated that CBZ increased water permeability and water absorption in the inner medullary collecting duct perfused in vitro. This effect was attributed to CBZ acting directly on the V2 vasopressin receptor (V2R) G protein-coupled receptors (GPCRs) complex and increasing the expression of aquaporin 2 (AQP2) [28].

Further understanding of the intracellular mechanisms involved in CBZ-induced hyponatremia comes from investigations by Sekiya and Awazu, and Kim et al. [7,29]. Sekiya et al. showed a patient who developed hyponatremia due to CBZ administration, noting suppressed plasma AVP levels and high urine cyclic adenosine monophosphate (cAMP)/osmolality, highlighting the cAMP-dependent action of CBZ via the V2 receptor on collecting ducts [29]. Kim et al. delved into the intracellular mechanisms of CBZ-induced AQP2 upregulation in the kidney, concluding that CBZ acts as a V2 receptor agonist, leading to aquaporin upregulation by accelerating AQP2 transcription and dephosphorylation [7].

Risk factors

While many cases of ASM-induced hyponatremia are asymptomatic and do not require intervention, severe hyponatremia (defined as a sodium level <128 mEq/L in <48 hours) can lead to neurological damage, seizures, altered mental status, brainstem herniation, and even death, the severity of these consequences underscores the importance of identifying risk factors associated with ASM-induced hyponatremia to reduce patient morbidity and mortality [30].

In a comprehensive twelve-year large-scale cross-sectional cohort study conducted by Yamamoto et al., certain ASMs were identified to be more frequently associated with severe hyponatremia [31]. Additionally, the study highlighted that the use of multiple ASMs, specific drug combinations, and independent patient risk factors contribute to an increased risk of severe hyponatremia. For instance, the study found that sodium levels decreased as the dose of CBZ increased, with a significant 10.9-fold higher prevalence of hyponatremia at CBZ doses exceeding 600 mg/day. This risk was further potentiated by the concomitant use of valproate [31].

Similarly, as Kim et al. demonstrated, the co-administration of OXC with other ASMs increased the risk of severe-range hyponatremia (P = 0.043). Moreover, patients taking OXC with non-ASM drugs also led to a significant increase in the risk of developing OXC-induced severe (P < 0.001) and symptomatic (P = 0.002) hyponatremia. The study highlighted that the use of non-steroidal anti-inflammatory drugs (NSAIDs), calcium channel blockers (CCBs), tricyclic antidepressants (TCAs), and diuretics led to an increased risk for the development of severe range hyponatremia. Old age and the use of diuretics were identified as crucial independent patient risk factors for OXC-induced severe and symptomatic hyponatremia [30].

A population cohort study conducted in Ontario, Canada, further demonstrated that advancing age increases the risk of severe range hyponatremia, with increasing age and CBZ use versus nonuse was associated with a higher 30-day risk of hospitalization with hyponatremia: 82/21,191 (0.39%) versus 30/63,573 (0.05%) with the relative risk (RR) being 8.20 with a 95% confidence interval (CI) 5.40-12.46 (16).

Gender differences also emerged as a notable factor in ASM-induced hyponatremia. Grikiniene et al. pointed out that females are more prone to ASM-induced hyponatremia compared to their male counterparts. This gender-related susceptibility is attributed to differences in sodium metabolism, sodium transport through the cell membrane, intracellular sodium concentration, and urinary sodium excretion [32].

Clinical symptoms and presentation

Mild hyponatremia is defined as a sodium level between 130-135 mEq/L or a gradual decrease in sodium >48 hours and is usually associated with minimal symptoms. Moderate hyponatremia is a sodium level between 125-129 mEq/L and can be associated with headache, fatigue, anorexia, and muscle cramping. As mentioned above, severe hyponatremia is defined by a sodium level <128 mEq/L that results in <48 hours or a sodium level <120 mEq/L regardless of the time it developed and is associated with the most serious symptoms, including coma [33]. 

In a study by Falhammar et al. of the common ASMs prescribed, CBZ, OXC, and levetiracetam were found to affect sodium levels with the highest incidence [34]. The actual prevalence of ASM-induced hyponatremia is challenging to ascertain due in part to many patients not being identified, as this condition is often clinically silent. Asymptomatic hyponatremia can persist until sodium levels are significantly low, and subtle symptoms may include headache, irritability, nausea, vomiting, and mental disorientation.

The consequences of hyponatremia become more pronounced as sodium concentrations decrease, potentially leading to increased seizure frequency, respiratory issues, ataxia, and even coma due to cerebral edema [35,36]. In hyponatremia, free water travels from the hypo-osmolar extracellular fluid to the hyper-osmolar interior of the cell. Consequently, this can result in cerebral edema, especially in the acute setting. In acute hyponatremia, free water moves from the hypo-osmolar extracellular fluid to the hyper-osmolar interior of the cell, causing the cell to swell, contributing to cerebral edema. However, chronic hyponatremia allows for adaptation over time, allowing the brain cells to tolerate lower sodium levels without causing cerebral edema, creating a new osmotic equilibrium to prevent cell flooding. This adaptation explains why a significant number of patients with chronic hyponatremia remain asymptomatic [9].

Age emerges as a significant factor influencing the impact and severity of long-term hyponatremia. Drug-induced hyponatremia caused by drugs other than ASMs is also more commonly seen in the elderly often due to polypharmacy and self-medication in this population. However, important age-related physiological changes also affect the severity of the symptoms, including the body's reduced ability for sodium retention. The affected sodium homeostasis and the body's handling of ADH account for increased age as a significant risk factor [37]. The elderly population, in particular, experiences higher rates of hospitalization compared to their younger counterparts, even with similar degrees of hyponatremia. A study by Renneboog et al. highlights that elderly patients with chronic hyponatremia are more prone to falls, emphasizing the broader health implications of this condition in older individuals [36]. Additionally, a retrospective study by Kinsella et al. showed that there is an increased risk of fracture in older people with even mild hyponatremia [38].

Chronic hyponatremia induced by ASM may even have some degree of influence over the subjective side effects of the offending medication. In a study by Berghuis et al., 65% of the study group that complained of adverse effects had clinical hyponatremia compared to 21% with eunatremia. Moreover, the more significant the hyponatremia, the more reported adverse effects. Among the patients with adverse effects, 83% had severe hyponatremia compared to 55% with mild hyponatremia [39]. In another case-control study by Refardt et al., the findings demonstrated that mild but chronic hyponatremia was associated with neurocognitive deficits that improved after electrolyte replacement [40].

Treatment strategies

ASMs are believed to induce hyponatremia by causing a syndrome of inappropriate ADH secretion (SIADH). Therefore, treatment of hyponatremia is focused on the same strategies as treating SIADH with the addition of removing the offending medication. If hyponatremia is not considered severe and asymptomatic, removing the offending medication that is causing hyponatremia will suffice along with repeat (Na+) level measurement.

Other supportive measures for hyponatremia from any cause include fluid restriction and increasing salt intake via dietary adjustment or implementing salt tablets. If a patient has hyponatremia that leads to hospitalization, it is important to determine whether a patient has acute versus chronic hyponatremia. This allows the determination of the best method to correct sodium levels while simultaneously preventing the serious and feared complication of osmotic demyelination.

Osmotic demyelination is a serious neurological disorder that can develop from the rapid correction of hyponatremia. The cause is multifactorial and risk factors such as alcoholism, malnutrition, cirrhosis, and increased age can make a patient more prone to developing osmotic demyelination syndrome. Osmotic demyelination syndrome is the demyelination of the astrocytes in the pons leading to central pontine myelinolysis (CPM). This results from the hypo-osmotic environment of the bloodstream causing sodium to leave the cells rapidly. 

If it is determined that hyponatremia has been present for >48 hours, correction is acceptable but should still be done carefully and as slowly as reasonably possible. Even within the 48-hour window, case reports are proving osmotic demyelination syndrome can occur, albeit less likely than with chronic hyponatremia [41]. In the case of acute hyponatremia, cells in the brain have yet to become adjusted to the hyponatremic environment. Thus, prompt administration of supplemental sodium would correct the extracellular osmolality. If, however, it is known that hyponatremia has been present for >48 hours or if it is unknown how long the patient has been hyponatremic, the pace of correction is important to prevent osmotic demyelination. This is a result of adaptation, where the cells in the brain have created a new osmotic equilibrium [9,41].

However, suppose too much sodium is given rapidly. In that case, the extracellular fluid becomes hyperosmotic, and water is drawn from brain cells to reach equilibrium again, which then causes the syndrome of osmotic demyelination. Conversely, if the patient is already demonstrating neurologic symptoms, the patient is likely suffering from cerebral edema. In this situation, it is important to rapidly raise sodium levels to a safe level without regard for osmotic demyelination and then slow down once neurologic symptoms have improved. If there are signs of cerebral edema, the first management line is rapid correction with 3% saline at a rate of 1 mL/kg/hr for several hours. If more severe signs are present, such as seizures or brain herniation, the rate should be increased to 2-3 mL/kg/hr. Hypertonic saline should be stopped once severe symptoms have resolved and switched to normal saline as soon as feasible.

The goal of correction is no more than 10-12 mmol/L in 24 hours. In brief, if a patient’s ASM is causing hyponatremia, the best initial treatment is to switch to an alternate ASM. If the patient has symptoms of cerebral edema, the patient should be hospitalized and corrected rapidly or slowly based on acute versus chronic hyponatremia [9].

Discussion

Hyponatremia is a serious side effect of many ASMs. Hyponatremia is associated with increased length of hospital stays and a higher mortality in hospitalized patients. OXC, CBZ, and ESL are associated with higher rates of hyponatremia. Reported Incidence of hyponatremia ranges from 25-75% for OXC and 5-40% for CBZ. The risk of hyponatremia with valproic acid, phenytoin, and topiramate is less understood and documented. The mechanism of ASMs-induced hyponatremia was shown to be due to an increased sensitivity to circulating AVP. Stephens et al. found that OXC affects renal collecting tubules directly and enhances their responsiveness to circulating AVP [8].

De Braganca et al. found that CBZ can increase water permeability in the medullary collecting duct, which induces hyponatremia via dilution [28]. Before this, hyponatremia from ASMs has been mostly attributed to SIADH. Various risk factors increase the chance of developing hyponatremia with ASM use. The simultaneous use of medications such as NSAIDs, TCAs, CCBs, and diuretics was found to increase this risk. Old age was also shown to be an important variable. Of these, old age and the contaminant use of diuretics were the most important independent risk factors. ASMs induce chronic hyponatremia that becomes clinically significant at levels <128 mEq. The associated findings can include headache, irritability, nausea and vomiting, and mental disorientation. Once the sodium concentration reaches low enough levels, it can increase seizure frequency and cause respiratory issues, ataxia, and even coma as a result of cerebral edema (6). Elderly patients are at higher risk of hospitalization and more severe symptoms than their younger counterparts, even at similar sodium levels.

Treatment options for ASM-induced hyponatremia vary depending on the degree of severity and the offending medication. Stopping the medication is an option for severe cases, especially if they are refractory to other treatments. The risk versus reward method should be used in patients treated with ASMs. Switching the ASM to another medication in the same category is another option. Other options include fluid resuscitation, fluid restriction, and increasing dietary sodium intake. It is important to note that ASMs are often a necessary entity and the treatment of a patient's seizures is often the ultimate goal. Therefore, if the patient's seizures are controlled by an ASM causing their hyponatremia and switching the ASM is not an option, management of the hyponatremia along with continuation of the ASM is necessary. 

## Conclusions

It is important to determine if the hyponatremia is acute or chronic when deciding on treatment methods. Complications such as osmotic demyelination are seen with rapid correction of chronic hyponatremia and should be avoided by titration, slowly bringing sodium levels back to a normal range. Acute hyponatremia responds better to prompt correction of sodium level. If neurologic symptoms are already present, cerebral edema needs to be considered as a complication of the low sodium level. If cerebral edema is found, you must raise sodium levels rapidly without consideration of osmotic demyelination. Hyponatremia is a very common electrolyte disorder that needs to be treated if identified. Hyponatremia can be a potentially fatal complication of ASMs. Healthcare must be aware of hyponatremia as a side effect of ASMs, as well as the signs and treatment options for the condition.

## References

[1] Adrogué HJ, Tucker BM, Madias NE (2022). Diagnosis and management of hyponatremia: a review. JAMA.

[2] (2024). Prevalence and risk factors for hyponatremia in adult epilepsy patients: large-scale cross-sectional cohort study. https://www.clinicalkey.com/.

[3] Cascade E, Kalali AH, Weisler RH (2008). Varying uses of anticonvulsant medications. Psychiatry (Edgmont).

[4] Abou-Khalil BW (2022). Update on anti seizure medications. Continuum (Minneap Minn).

[5] Davies JA (1995). Mechanisms of action of antiepileptic drugs. Seizure.

[6] Asconapé JJ (2002). Some common issues in the use of antiepileptic drugs. Semin Neurol.

[7] Kim GH (2022). Pathophysiology of drug-induced hyponatremia. J Clin Med.

[8] Stephens WP, Coe JY, Baylis PH (1978). Plasma arginine vasopressin concentrations and antidiuretic action of carbamazepine. Br Med J.

[9] Hoorn EJ, Zietse R (2017). Diagnosis and treatment of hyponatremia: compilation of the guidelines. J Am Soc Nephrol.

[10] Rho JM, White HS (2018). Brief history of anti-seizure drug development. Epilepsia Open.

[11] Mutanana N, Tsvere M, Chiweshe MK (2020). General side effects and challenges associated with anti-epilepsy medication: a review of related literature. Afr J Prim Health Care Fam Med.

[12] Battino D, Tomson T, Bonizzoni E (2024). Risk of major congenital malformations and exposure to antiseizure medication monotherapy. JAMA Neurol.

[13] Lu X, Wang X (2017). Hyponatremia induced by antiepileptic drugs in patients with epilepsy. Expert Opin Drug Saf.

[14] Gupta DK, Bhoi SK, Kalita J, Misra UK (2015). Hyponatremia following esclicarbazepine therapy. Seizure.

[15] Chung S, Sinha SR, Shah A (2019). Long-term safety and efficacy following conversion to eslicarbazepine acetate monotherapy in adults with focal seizures. Epilepsy Res.

[16] Gandhi S, McArthur E, Mamdani MM (2016). Antiepileptic drugs and hyponatremia in older adults: two population-based cohort studies. Epilepsia.

[17] Pendlebury SC, Moses DK, Eadie MJ (1989). Hyponatraemia during oxcarbazepine therapy. Hum Toxicol.

[18] Johannessen AC, Nielsen OA (1987). Hyponatremia induced by oxcarbazepine. Epilepsy Res.

[19] Friis ML, Kristensen O, Boas J (1993). Therapeutic experiences with 947 epileptic out-patients in oxcarbazepine treatment. Acta Neurol Scand.

[20] Dong X, Leppik IE, White J, Rarick J (2005). Hyponatremia from oxcarbazepine and carbamazepine. Neurology.

[21] Berghuis B, de Haan GJ, van den Broek MP, Sander JW, Lindhout D, Koeleman BP (2016). Epidemiology, pathophysiology and putative genetic basis of carbamazepine- and oxcarbazepine-induced hyponatremia. Eur J Neurol.

[22] Intravooth T, Staack AM, Juerges K, Stockinger J, Steinhoff BJ (2018). Antiepileptic drugs-induced hyponatremia: review and analysis of 560 hospitalized patients. Epilepsy Res.

[23] Perucca E, Richens A (1980). Reversal by phenytoin of carbamazepine-induced water intoxication: a pharmacokinetic interaction. J Neurol Neurosurg Psychiatry.

[24] Nasrallah K, Silver B (2005). Hyponatremia associated with repeated use of levetiracetam. Epilepsia.

[25] Rosca EC, Simu M (2018). Levetiracetam-induced hyponatremia. Acta Neurol Belg.

[26] Feldman BJ, Rosenthal SM, Vargas GA (2005). Nephrogenic syndrome of inappropriate antidiuresis. N Engl J Med.

[27] Sachdeo RC, Wasserstein A, Mesenbrink PJ, D'Souza J (2002). Effects of oxcarbazepine on sodium concentration and water handling. Ann Neurol.

[28] de Bragança AC, Moyses ZP, Magaldi AJ (2010). Carbamazepine can induce kidney water absorption by increasing aquaporin 2 expression. Nephrol Dial Transplant.

[29] Sekiya N, Awazu M (2018). A case of nephrogenic syndrome of inappropriate antidiuresis caused by carbamazepine. CEN Case Rep.

[30] Kim YS, Kim DW, Jung KH (2014). Frequency of and risk factors for oxcarbazepine-induced severe and symptomatic hyponatremia. Seizure.

[31] Yamamoto Y, Takahashi Y, Imai K, Ohta A, Kagawa Y, Inoue Y (2019). Prevalence and risk factors for hyponatremia in adult epilepsy patients: Large-scale cross-sectional cohort study. Seizure.

[32] Grikiniene J, Volbekas V, Stakisaitis D (2004). Gender differences of sodium metabolism and hyponatremia as an adverse drug effect. Medicina (Kaunas).

[33] Rondon H, Badireddy M. (2024). Hyponatremia. StatPearls [Internet].

[34] Falhammar H, Lindh JD, Calissendorff J, Farmand S, Skov J, Nathanson D, Mannheimer B (2018). Differences in associations of antiepileptic drugs and hospitalization due to hyponatremia: a population-based case-control study. Seizure.

[35] Čiauškaitė J, Gelžinienė G, Jurkevičienė G (2022). Oxcarbazepine and Hyponatremia. Medicina (Kaunas).

[36] Yamamoto Y, Ohta A, Usui N, Imai K, Kagawa Y, Takahashi Y (2023). Incidence trends and risk factors for hyponatremia in epilepsy patients: a large-scale real-world data study. Heliyon.

[37] Renneboog B, Musch W, Vandemergel X, Manto MU, Decaux G (2006). Mild chronic hyponatremia is associated with falls, unsteadiness, and attention deficits. Am J Med.

[38] Kinsella S, Moran S, Sullivan MO, Molloy MG, Eustace JA (2010). Hyponatremia independent of osteoporosis is associated with fracture occurrence. Clin J Am Soc Nephrol.

[39] Berghuis B, Hulst J, Sonsma A (2021). Symptomatology of carbamazepine- and oxcarbazepine-induced hyponatremia in people with epilepsy. Epilepsia.

[40] Refardt J, Kling B, Krausert K, Fassnacht M, von Felten S, Christ-Crain M, Fenske W (2018). Impact of chronic hyponatremia on neurocognitive and neuromuscular function. Eur J Clin Invest.

[41] Hwang C (2023). Osmotic demyelination syndrome in a high-risk patient despite cautious correction of hyponatremia. Electrolyte Blood Press.

